# Hypotension and antiphlogistic potential of empagliflozin ocular film: swelling and release kinetics

**DOI:** 10.5599/admet.2941

**Published:** 2025-11-24

**Authors:** Tanisha Das, Subrata Mallick, Sourajit Parida, Mouli Das, Rakesh Swain, Sk Habibullah

**Affiliations:** School of Pharmaceutical Sciences, Siksha ‘O’ Anusandhan (Deemed to be University), Bhubaneswar, Odisha, India

**Keywords:** SGLT2 inhibitor, hydration dynamics, drug dissolution, IOP, ocular anti-inflammation, SiO_2_

## Abstract

**Background and Purpose:**

Empagliflozin (EMP) is a sodium-glucose cotransporter 2 (SGLT2) inhibitor used for the management of type 2 diabetes mellitus. The danger of glaucoma in type 2 diabetes mellitus patients is known to be reduced using SGLT2 inhibitors. Empagliflozin is also believed to reduce the level of inflammatory markers. The present work has been undertaken for monitoring intraocular pressure and anti-inflammatory activity using the empagliflozin ocular film formulation. The effect of colloidal silicon dioxide upon the dynamics of swelling and drug release performance was also studied.

**Experimental Approach:**

Hydroxypropyl methylcellulose-based ocular film of empagliflozin was prepared, including silicon dioxide in different ratios as 1:0.00, 1:0.01, 1:0.02, 1:0.04, and 1:0.06 (namely, EMA0, EMA1, EMA2, EMA3 and EMA4, respectively) using casting and solvent evaporation. Swelling and drug release studies of the films were conducted in phosphate buffer solution (pH 7.4), and the kinetic mechanisms of swelling and drug release were evaluated. Intraocular pressure was measured after application of the film in the normotensive rabbit eye. Moreover, the anti-inflammatory effect was assessed using a rabbit ocular carrageenan-induced inflammation model.

**Key Results:**

Swelling behaviour followed Fickian mechanism in the order: EMA3<EMA2<EMA0<EMA4<EMA1, and partial relaxation with EMA3. Films containing silicon dioxide showed faster release than those without it (EMA0), following a diffusion pattern. The silicon dioxide-loaded film (EMA3) showed significantly lowered intraocular pressure and promising ocular anti-inflammation with favourable binding affinities of EMP to Interleukin-1β, Interleukin 6 and tumour necrosis factor-α. A good correlation between intraocular pressure and drug release was also established.

**Conclusion:**

A hydroxypropyl methylcellulose-based ocular film containing empagliflozin and silicon dioxide could be used to manage intraocular pressure and inflammation in a controlled manner in patients with type 2 diabetes mellitus.

## Introduction

Empagliflozin (EMP), the sodium-glucose co-transporter 2 (SGLT2) inhibitor, is used in the control of the glucose level of a person with type-2 diabetes mellitus (T2DM) [1]. SGLT2 inhibitor, in general, is recognized to have beneficial effects on both systolic and diastolic blood pressure, as well as lowering the risk of cardiovascular disease [2,3]. Moreover, the risk of glaucoma in T2DM patients is acknowledged to be reduced using SGLT2 inhibitors rather than glucagon-like peptide-1 receptor agonists and dipeptidyl peptidase-4 inhibitors [2,3].

EMP also plays an important role as an antiphlogistic agent by blocking the induction of brain-derived neurotrophic factor (BDNF), which occurs by reducing the production of neuro-inflammatory markers, tumour necrosis factor-α (TNF-α), and interleukin 1β (IL-1β) [4]. The systemic anti-inflammatory effect of EMP has been demonstrated recently [5].

An EMP hydrogel-forming ocular film formulation was developed incorporating colloidal silicon dioxide to avoid rapid clearance, ensuring sustained, controlled and targeted delivery of the drug, increasing residence time. Ocular hypotension and anti-inflammatory activity have been monitored using EMP film topically. Swelling performance after hydration and release was evaluated to understand the kinetic behaviour. Hydration and swelling of the polymer significantly impact polymer chain relaxation, enabling drug release patterns in a controlled manner [6]. It also enhances bio-adhesion by promoting interface contact between the polymer and mucosal tissue. Mucosal administration majorly depends not only on the drug release kinetics but also on the interaction of the delivery system with the mucosa, clearance rate, and time, as the polymer chains intertwine with the mucin chains of the mucosal membrane [7]. Enhanced swelling properties result in an increase in the pore size of the polymeric ocular film, thereby facilitating the diffusion of the active agent. The ocular hydration property improves with increasing film swelling index, resulting in a slow erosion profile.

Silica in nano form is known to be used as the most encouraging ocular drug delivery carrier [8]. Silica acts as a permeation enhancer in the ocular delivery of drugs [8,9]. Microtubule-associated protein 1 light chain 3 (MAP1-LC3) A, B, and C (LC3s) are structural proteins of autophagosomal membranes, widely used as biomarkers of autophagy. Increased LC3A/B-II expression, resulting from the cellular uptake of silica in nanoform, activates autophagy and is responsible for reducing ocular inflammation. Consequently, mammalian target of rapamycin (mTOR) is activated within the corneal epithelium and decreases the progression of diabetic retinopathy [8]. Colloidal SiO_2_ biologically degrades into various protonated ortho-silicate ion forms after intravitreal injection due to the polycondensation of internal silanol groups [10,11]. These byproducts are efficiently cleared from the aqueous and vitreous humour through normal turnover processes.

Empagliflozin ocular film using Hydroxypropyl methylcellulose (HPMC) matrix polymer was prepared, and the effect of colloidal silicon dioxide has been studied on hydration and drug release. Ocular hypotension and antiphlogistic activity have also been examined. The prepared ocular film is anticipated to exhibit a promising level of hydration and significant drug release in the presence of SiO_2_ at the nano-level and reduce the ocular pressure significantly compared to the film without SiO_2_, when applied topically.

## Experimental

### Materials

EMP was collected from YARROW CHEM, Mumbai, India. Hydroxypropyl methylcellulose K15 (HPMC: METHOCEL K15M, ID34709, Lot No.: GAR492084, Viscosity: 12,000-18,000 cP (2 % in water at 20 °C), Colorcon Asia Private Limited, India) received as gift sample, and Colloidal silicon dioxide (Aerosil 200; CAS No.: 112945--52-5) was acquired from Hi-media laboratories (Nashik, India). Polyethylene glycol 400 (PEG; grade: ExiPlus, multi-compendial; CAS No.: 25322-68-3; viscosity: 120 mPa s; density: 1.126 g mL^-1^) was obtained from Sisco Research Laboratories, Mumbai, India. Other chemicals obtained were of laboratory grade.

### Preparation of ocular film

Initially, the dispersion was prepared in distilled water (100 mL) in a volumetric flask using a weighed amount of SiO_2_ and stirred magnetically overnight and finally ultrasonicated (Digital Ultrasonic Cleaner-CD-4820, Capacity 2500 ml, 170 W, Codyson Electrical Co., Ltd., China) for 1 h. The polymeric base gel was prepared by dispersing HPMC uniformly in about 40 mL of distilled water after overnight hydration. An aliquot of the SiO_2_ dispersion was pipetted out into the polymeric base gel. Ethanolic solution of EMP (100 mg in 10 mL) and PEG were added to the base gel and stirring was continued magnetically for homogeneous distribution for about 8 h. Drug : SiO_2_ weight ratios as 1:0.00, 1:0.01, 1:0.02, 1:0.04 and 1:0.06 were used for film formulations (EMA0, EMA1, EMA2, EMA3 and EMA4 respectively) as tabulated in Table 1. After casting in a petri dish (diameter: 90 mm), the petri dish was placed in a hot air oven (about 40 °C) for drying (about 48 hours) to constant weight [12].

**Table 1.** Empagliflozin film formulation incorporating colloidal silicon dioxide and physicochemical properties for ocular delivery (mean ± SD; n = 3 or more), HPMC content 1200 mg, PEG content 20 % with respect to the amount of HPMC as matrix polymer, folding endurance >200

Film code	Drug: SiO_2_	Thickness, μm	Surface pH	Erosion, %^a^
EMA0	1 : 0.00	257.5 ± 13.5	7.29 ± 0.05	38.23 ± 0.34
EMA1	1 : 0.01	263.0 ± 2.6	7.31 ± 0.05	12.79 ± 1.49
EMA2	1 : 0.02	287.0 ± 17.6	7.33 ± 0.08	54.27 ± 3.12
EMA3	1 : 0.04	337.5 ± 9.2	7.34 ± 0.06	87.83 ± 6.65
EMA4	1 : 0.06	339.6 ± 25.7	7.35 ± 0.06	61.51 ± 1.66

^a^Loss of soluble material from the film into the fluid in liquid environment at 3 h.

### Physicochemical characterization

The thickness of the prepared film (about 2×2 cm) was measured using a Digital Micrometer (Mitutoyo, Japan) across different regions of the entire film. The surface pH of the film was determined by adding a small amount of PBS 7.4 dropwise onto the surface of a small piece (about 2×2 cm) of the polymeric film. Then, the probe of the pH meter (Systronics Digital pH meter with Electrode 335; 1 mV resolution; pH range: 0 to 14; readout: 3 ½ digit LED) was brought in contact with the hydrated surface of the film piece tillae constant reading was obtained. The folding endurance of the film was also assessed to ensure the durability of the film by folding it on the same line continuously till breakage. This physicochemical parameters assessment was conducted in triplicate or more and results were recorded as mean ± standard deviation (SD) [13].

### Swelling and matrix erosion study

The swelling and erosion behaviour of the film formulation was evaluated based on hydration and matrix erosion. Initially, a glass slide containing an accurately weighed (*W*_1_) piece of film (about 2×2 cm) was placed in a petri dish (90 mm diameter) containing 25 mL of PBS, pH 7.4, at room temperature. At regular time intervals (10, 20, 30, 45, 60, 90, 120, 150 and 180 min), the slide was removed and re-weighed (*W*_2_). Every time, the excess PBS on the surface of the film was carefully wiped off with tissue paper on the swollen surface. This procedure was implemented three times or more for each film and presented as mean ± SD. The film was then allowed to dry at 60 °C for 24 hours and stored in desiccators for over 48 hours. After drying, the film's weight was again recorded (*W*_3_).

The hydration, % or swelling, and matrix erosion, % were calculated by using Equations (1) and (2):



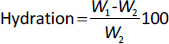

(1)




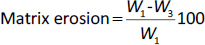

(2)


### Fourier transform infrared spectroscopy study

The pure EMP and film formulations were scanned over 4000 to 600 cm^-1^ at a rate of 16 scans per 4 cm to study drug-excipient interactions. The scans were performed in the range of 4000 to 600 cm^-1^ to interpret the IR transmission spectra using the attenuated total reflectance - Fourier Transform Infrared (ATR-FTIR) spectrophotometer (Jasco FT/IR 4600) by placing the samples on the diamond ATR (JASCO ATR PRO ONE) crystal [14].

### X-ray diffraction (XRD) study

X-ray crystallographic studies of EMP and prepared films were performed using a powder X-ray diffractometer (P-XRD; Model: Rigaku Ultima IV) with Cu X-rays (*λ* = 0.154 nm). The scans were performed at 1° per minute to record the value of 2*ϑ* in the range of 5 to 65° [13].

### Differential scanning calorimetry

The thermal behaviour of the drug alone and the films (EMA0-EMA4) was studied using a differential scanning calorimetry instrument (DSC, Mettler Toledo; Software-Star E, SNR-18289) under a purged nitrogen environment (flow rate: 50 mL min-1) to ensure that the drug and the excipients remained inert during interaction. The temperature was allowed to increase at a speed of 10 °C min^-1^ in the range of 30 to 250 °C [14].

### Scanning electron microscopy

The surface morphology of the pure drug and the films was investigated using scanning electron microscopy (SEM; Model: ZEISS, EVO 18) at different magnifications of 1,000 to 20,000 times. A voltage of 5 to 15 kV was applied to capture the photomicrographs for morphological assessment of the samples [14].

### In-vitro drug release study

USP type II apparatus (Electro Lab, TDT-06L India) was used to study *in vitro* drug release [12]. Dissolution testing was performed in medium (200 mL; PBS, pH 7.4) at 34 ± 2 °C and 50 rpm. A strip cut of film (20 mg equivalent drug) was accurately weighed, attached to a glass slide with cyanoacrylate adhesive, and placed at the bottom of the dissolution vessel containing 200 mL of medium. At a predetermined time interval, a 10 mL sample was collected and replaced with fresh medium. The sample was analysed spectrophotometrically at a wavelength of 224 nm using a UV-visible spectrophotometer (Shimadzu UV-1900i), and the data were recorded as mean ± SD of three or more replicates.

### Mechanism of swelling and drug release kinetics

The estimated swelling and *in vitro* drug release data were allowed to fit in both the Korsmeyer-Peppas and Peppas-Sahlin models for understanding the mechanism of swelling and drug release kinetics by using Equations (3) and (4) [15]:

Korsmeyer-Peppas model:



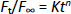

(3)




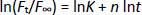

(4)


where *F_t_*/F is *the* fraction of drug release at time *t, K* is the Peppas release rate constant, and n is the exponent of swelling/release/permeation

Peppas Sahlin model, Equation (5):



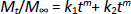

(5)


where *k*_1_ is the Fickian diffusion constant of swelling /release/permeation kinetics, *k*_2_ is the relaxation kinetics constant, and *m* is the Peppas Sahlin exponent of diffusion.

The estimation of the n value of the Korsmeyer Peppas model was done, and *k*_1_, *k*_2_ and *m* values of the Peppas-Sahlin model were estimated for both swelling and drug release kinetics.

### Sterility studies

The film formulation (EMA3) was sterilized initially by UV exposure at a distance of 25 cm on both sides for 10 min [13]. Sterility testing of the hydrogel film formulation (EMA3) was performed following the USP guidelines. A 100 mg sample of sterilized ocular film was aseptically transferred into sterile test tubes containing 10 mL of culture media. Microbial strains, *Staphylococcus aureus* (MTCC 96) were selected for the study. For bacterial strain evaluation, two media were used: Soybean-casein digest medium and Fluid Thioglycollate medium. All media were prepared and sterilized according to standard microbiological procedures [16]. The positive control consisted of 10 mL of sterile media inoculated with 0.1 mL of microbial suspension. The microbial suspensions were prepared by culturing each organism in nutrient broth and adjusting the turbidity to match the 0.5 McFarland standard (~1.5×10^8^ CFU mL^-1^). Serial dilutions were performed to achieve a working concentration of approximately 100 CFU mL^-1^. The negative control contained only sterile media to confirm its sterility. The test samples consisted of film (EMA3) and media and were not inoculated with any microbial suspension. All tubes were incubated for 14 days under appropriate conditions, acclimatizing the bacterial samples at 37 ± 2 °C. Observations were recorded for signs of turbidity and/or visual microbial growth on specific days (day 1, day 3, day 7 and day 14).

### In vivo animal studies

A normotensive healthy New Zealand rabbit model (2 to 2.5 kg) free from any clinical ocular abnormalities was used for in vivo assessments. The rabbits were allowed to acclimatize within the laboratory setting prior to the commencement of the experiment [17]. The animal experiment was approved by the Institutional Animal Ethical Committee (IAEC), bearing registration number IAEC/SPS/SOA/124/2022. The animal handling procedure was carried out by following the guidelines of CPCSEA (Committee for the Purpose of Control and Supervision of Experiments on Animals) [18] followed by the compliance with ARRIVE (animal research reporting of *in vivo* experiments) principles [19]. The animals were housed in 12-hour light and dark intervals, maintaining an air-conditioned environment (temperature: around 25 °C, relative humidity (RH) 50 % approx.), offering water and food *ad libitum* [13].

Following the Institutional Animal Ethics Committee (IAEC) protocol, the animals were allocated into 3 groups to study the effect of EMP ocular film loaded with SiO_2_ (EMA3) and without SiO_2_ (EMA0) on the normal intraocular pressure (IOP), respectively. Group I consisted of rabbits that received no treatment. Group II included the rabbits induced with corneal inflammation using carrageenan injection in the upper palpebral region, and Group III comprised the rabbits treated with Ocusert formulations (EMA3 and EMA0, separately). They were individually housed in a properly air-conditioned and light-controlled room laboratory maintaining 25±1 °C and 70±5 % RH, followed by a standard pellet diet, and water *ad libitum*. The effect of empagliflozin on IOP was monitored using the Schiøtz Riester Tonometer (Germany) [13]. The rabbits were placed in laboratory hutches for IOP measurements. Half an hour before the experiment, the animals were anesthetized with 0.5 % tetracaine HCl (0.75 mg / 150 μL), and an initial value of IOP was recorded. One 1.5×2.5 mm half-moon-shaped film piece (pre-sterilized by UV exposure for 10 min) was placed in the cul-de-sac region of the rabbit eye, followed by IOP measurement at 30-minute time intervals till the IOP exhibited normal. The reading was recorded discreetly for EMA0 and EMA3, and the data were plotted graphically as a decrease in ocular pressure over time. The experiment was performed in triplicate and is therefore presented as the mean ± SD. Lastly, the rabbit eyes were properly rinsed with normal saline solution, followed by the administration of 0.5 % w/v moxifloxacin HCl eyedrops.

Similarly, to study ocular inflammation, healthy male New Zealand rabbits were used and grouped into three groups: Group I (test group with the formulation), Group II (positive control), and Group III (negative control). The positive control group was induced with corneal inflammation by injecting carrageenan (200 μl, 2 % w/v) into the upper palpebral region. After receiving carrageenan injection, a 1.5×2.5 mm film (EMA3) was placed in the lower cul-de-sac region of the test group rabbit. Ocular anti-inflammatory response was examined at specific time intervals by visual inspection in comparison with the negative control group (healthy rabbits). Proparacaine HCl injection was administered for local anaesthetic purposes. At the end of the experimental procedure, moxifloxacin HCl (0.5 % w/v) eye drop was instilled for a quick eye recovery.

### Correlation study

To understand the relationship and predictability, the correlation of swelling *vs.* drug release and the area under the activity curve (AUAC) of reduced ocular pressure versus drug release has been measured using MS Office (Excel) 2021. At the same time points for swelling and drug release, as well as for decrease in ocular pressure and drug release, correlation plots were constructed. The average trendline was recorded to determine the correlation coefficient (*r*^2^).

### Stability studies

The accelerated stability studies were carried out according to International Conference on Harmonization (ICH) Q1A (R2) guidelines [20]. The five ocular film formulations, along with pure EMP, were stored at 40 °C and 75 % RH for a period of 6 weeks in a stability chamber [21] to ensure the stability of ocular films. FTIR spectra of the ocular preparations were obtained after 6 weeks of storage and were compared with the initial spectra to determine changes in drug-excipient interactions (if any). Subsequently, DSC was also conducted with pure EMP and the film formulations (after 6 weeks of storage) to observe any changes in the heat flow vs. melting point plots.

### Docking study

The computational study was implemented in a DELL Inspiron 15 with an Intel Core i3 processor, having 8GB RAM and a 512 GB SSD. The UniProt and Protein Data Bank helped retrieve the receptor structures and ligands (empagliflozin and diclofenac) from the PubChem database in 3D SDF format. The active site of the receptor was predicted using the PrankWeb database [14]. A docking study was implemented between Empagliflozin with interleukin 1β (IL-1β), interleukin-6 (IL-6), and tumour necrosis factor-α (TNF-α) receptors, followed by diclofenac with the same receptors using Pyrex software. The optimum binding affinities of the ligands to the receptors were represented with the help of the unit kJ mol^-1^.

## Results and discussion

### Physicochemical parameter

Empagliflozin ocusert film formulation in the presence and absence of colloidal silicon dioxide exhibited a thickness of 225.5 ± 18.5 to 353 ± 41.5 μm, which is favourable for ocular delivery (Table 1). The folding endurance of all the formulations was well above 200, showing their flexibility and ruggedness [22]. The surface pH ranged between 7.2 and 7.4, which is convenient for ocular delivery without any irritation [12].

### Swelling and erosion study

**Figure 1.**
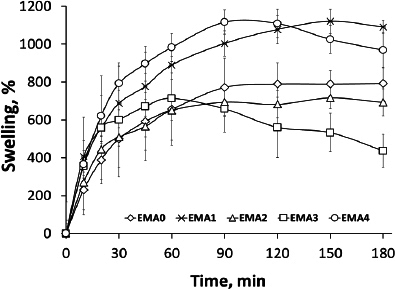
Hydration and swelling profile of the film in the presence and absence of silicon dioxide

Figure 1 shows the swelling *vs.* time profile of the formulations in the presence and absence of SiO_2_. The maximal levels of hydration and swelling were observed with EMA1 and EMA4 after 180 min (1091 % and 986 %, respectively) and later declined due to erosion. Whereas EMA2 and EMA3 exhibited a minimal level of hydration (693 and 437 %, respectively, at 180 min), declining after 60 to 90 min. A stagnantly maintained medium level of hydration was observed in the absence of SiO_2_ (EMA0) of 772 to 792 % (90 to 180 min). The main reason behind the swollen polymeric matrix is due to the presence of hydroxyl (-OH) groups, enabling water absorption via H-bonding, leading to an enhanced swelling index [23]. A higher swelling index was associated with lower matrix erosion, and the two were inversely related. The matrix erosion was found to be highest in EMA3 (~88 %) and least in EMA1 [12], as presented in Table 1.

### Fourier transform infrared spectroscopy study

**Figure 2.**
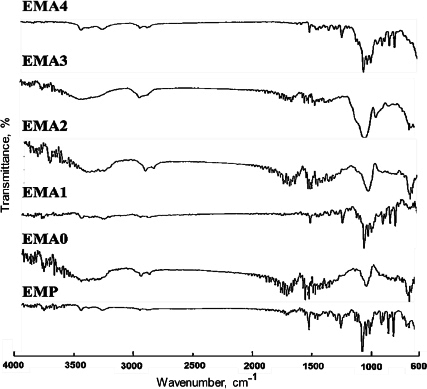
FTIR spectrum of the ocular film with and without silicon dioxide

Fourier transform infrared (FTIR) spectrum (Figure 2) was obtained in the range of 4000 to 600 cm^-1^.

EMP displayed the presence of an absorption band at 1061 cm^-1^ (C-O stretching), 3423 cm^-1^ (O-H stretching), and 1600 cm^-1^ (ketonic stretching, which is a part of the tetrahydrofuran ring) [24,25]. The band at 984 cm^-1^ indicated the presence of silanol in the prepared film containing colloidal SiO_2_ (EMA0-EMA4), representing the symmetric stretching of the Si-O vibration [26]. Another peak at 1172 cm^-1^ designated the presence of silica, dispersed in nano-form within the film [8]. The characteristic peaks in the FTIR spectra indicated that there was no interaction or change in the chemical structure of EMP, HPMC, or the plasticizers, confirming that the drug has been completely embedded in the polymer matrix.

### X-ray diffraction study

**Figure 3.**
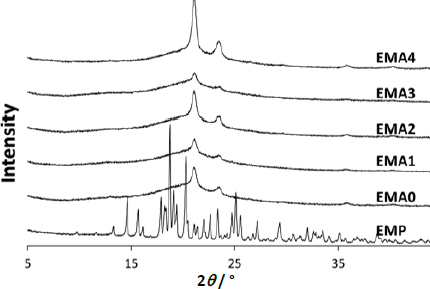
XRD of the pure drug (EMP) and ocular film formulation

The XRD analysis revealed that the pure drug (EMP), being crystalline, displayed distinct and sharp peaks in the diffractogram at 14.52, 18.64, 20.18 and 25.0°, indicating its well-organized molecular crystalline structure as displayed in Figure 3 [27]. Interaction among pure EMP, HPMC, and PEG resulted in almost complete amorphization of the drug in the formulation. The intensity of the crystalline drug peaks of the film containing hydrophilic polymers (HPMC and PEG), in the presence and absence of SiO2, diminished considerably, leading to disruption of the drug's crystal lattice. This is likely due to hydrogen bonding and molecular interactions between the -OH groups of the pure drug and the HPMC polymer network, followed by the solubilisation and plasticisation of PEG, as shown in Figure 3. Additionally, two prominent pointed peaks at 21.18 and 23.58° have been observed, potentially indicating some leftover ordered crystallinity (residual crystallinity) within PEG caused by temperature variations [28].

### Thermal analysis

**Figure 4.**
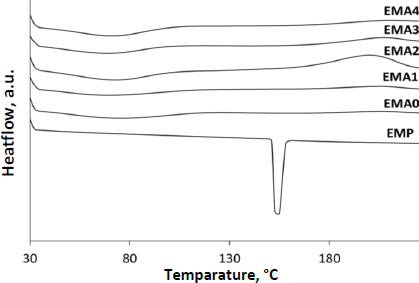
DSC thermogram of pure EMP and film formulation

DSC thermogram of pure drug (EMP) exhibited a melting endothermic peak at 152 °C. The endothermic peak disappeared in all the film preparations (Figure 4). Thermal analysis interpretation revealed the amorphization of the drug in the film [14]. The broadening of the endothermic peaks (50 to 80 °C) might be due to the evaporation of moisture entrapped in the polymeric matrix [14].

### Crystal morphological assessment

**Figure 5.**
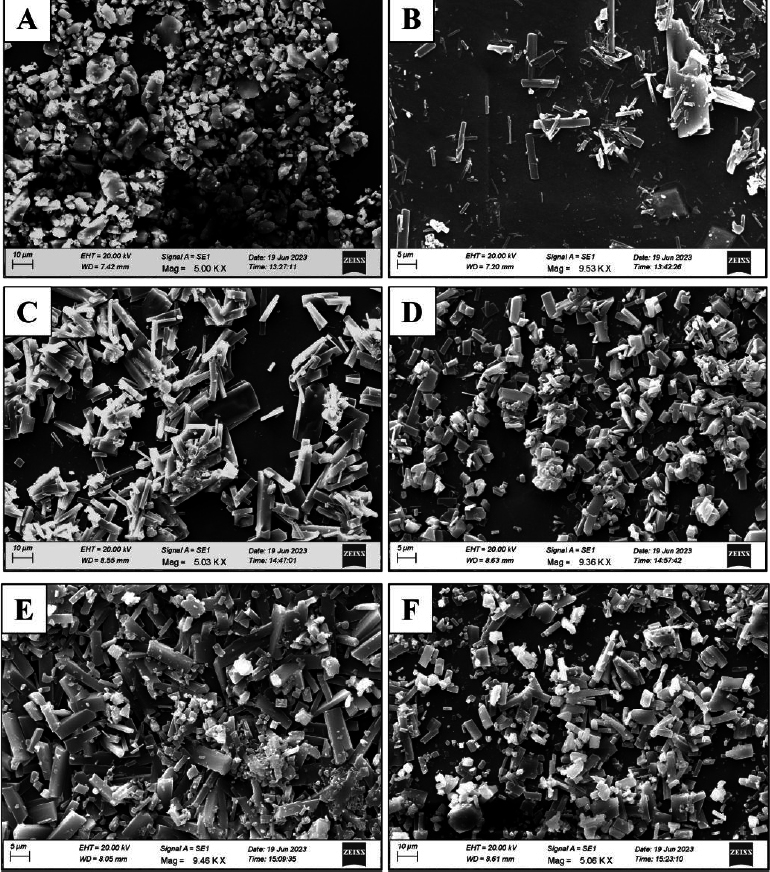
Crystal morphological study using SEM of pure drug and film formulation: (A) EMP, (B) EMA0, (C) EMA1, (D) EMA2, (E) EMA3, (F) EMA4

Figure 5 depicts the scanning electron microscopy of pure drug (EMP) and the film without and with SiO_2_. The geometry of the drug crystal was visible as shown in Figure 5A with a magnification of 5000×. Sharp rectangular-shaped crystal growth was observable in EMA0 as displayed in Figure 5B. The presence of SiO_2_ within the HPMC matrix significantly impacted the morphology and surface texture of the drug particles.

The pictographs exhibited good drug dispersion, effective hydration of the drug by the polymer, and strong adhesion within the drug-polymer system [29]. The drug crystal geometry exhibited amorphization, as evidenced by agglomerated crystallites in all formulations (EMA1 to EMA4), as shown in Figures 5C to 5F, and was further supported by pre-interpreted XRD and DSC studies. Drug diffused in the polymeric matrix mostly in EMA3 as compared to other formulations, as demonstrated in Figure 5E. However, the conducted SEM study exhibited a smooth surface of the ocular films, ensuring no fracture/cracks and uniform distribution of the drug within the film matrix [30].

### In-vitro drug release study

**Figure 6.**
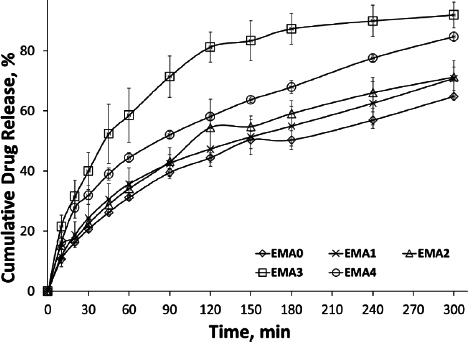
Cumulative drug release profile of the film without and with SiO_2_ (EMA0-EMA1)

All the film formulations were studied for *in vitro* release in PBS pH 7.4 (Figure 6). The fastest release was found in EMA3 (about 92 %), and the most delayed release was in EMA0 (about 48 %) at 300 min. All the film formulations containing SiO_2_ (EMA1-EMA4) showed faster release compared to the formulation without SiO_2_ (EMA0). The presence of internal -OH groups in the silanol of colloidal SiO_2_ facilitated adsorption, resulting in the delayed release of EMA4 (drug: SiO_2_ = 1: 0.06) compared to EMA3 [30]. The presence of SiO_2_ in the film improved the release (53 to 92 %) compared to the film without SiO_2_ (EMA0) at 300 min. The rate of drug release was found in the order of EMA0 < EMA1 < EMA2 < EMA3 < EMA4.

### Mechanism of swelling and drug release kinetics

After fitting the estimated swelling profile and cumulative drug release data to the Korsmeyer-Peppas and Peppas-Sahlin models, it was found that the observed values were significantly closer to the predicted values of the models. When the mechanism is found to be underexplored or exhibits more than one sort of possible swelling behaviour and drug release pattern, the Korsmeyer-Peppas model is generally employed to study the anomalous diffusion mechanism of polymeric dosage forms [31]. The significance of Peppas Sahlin is enabling the assessment of mathematical modelling of each drug release mechanism via certain constants, such as *k*_1_ and *k*_2_. If *k*_1_ is greater than *k*_2_, the drug release pattern is considered to follow Fickian diffusion. Consequently, if *k*_2_ is found to be greater than *k*_1_, relaxation of polymeric chains begins. The in vitro drug release profile demonstrated a diffusion mechanism in a controlled manner, exceeding 300 min (*k*_1_ > *k*_2_), as shown in Table 2.

**Table 2.** *In vitro* release kinetics and Swelling kinetics of the prepared ocular film formulations

Film Code	Swelling study					*In vitro* drug release study				
	Korsmeyer Peppas model		Peppas Sahlin model			Korsmeyer Peppas model		Peppas Sahlin model		
	*r* ^2^	*n*	*k*_1_	*k*_2_	*m*	*r*_2_	*n*	*k*_1_	*k*_2_	*m*
EMA0	0.9930	0.55	69.2	-1.47	0.63	0.9916	0.59	2.764	-0.027	0.62
EMA1	0.9922	0.53	116.4	-3.07	0.58	0.9956	0.58	3.979	-0.046	0.55
EMA2	0.9698	0.47	108.5	-4.10	0.52	0.9868	0.61	2.824	-0.027	0.65
EMA3	0.9758	0.46	199.7	-15.19	0.44	0.9947	0.52	4.821	-0.066	0.66
EMA4	0.9664	0.58	122.4	-3.40	0.60	0.9956	0.61	6.990	-0.081	0.47

**Figure 7.**
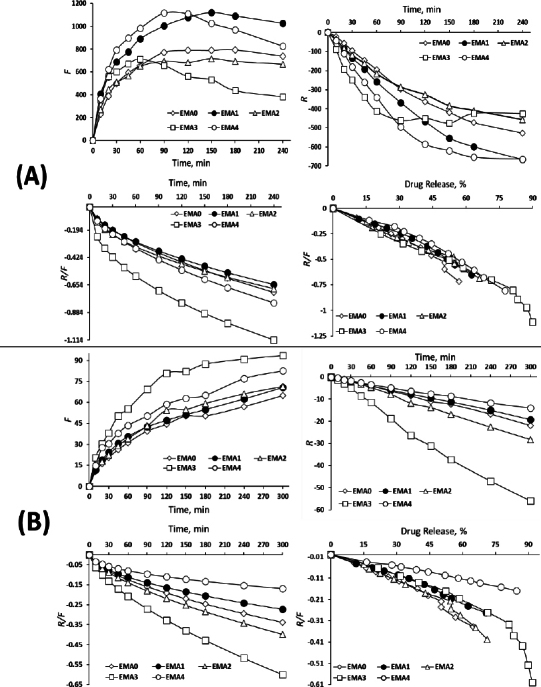
Fickian contribution, relaxation and relaxation over Fickian contribution plots for the estimation of (A) swelling kinetics, and (B) drug release kinetics

The swelling kinetics evaluated using the Korsmeyer-Peppas model yielded *n* values closer/nearer to 0.5, suggesting a predominantly Fickian diffusion mechanism. Complimentarily, Peppas-Sahlin model showed a higher rate constant for Fickian diffusion (*k*_1_) than that for polymer relaxation (*k*_2_), confirming the dominance of Case I diffusion across all formulations. However, the relaxation (*R*) versus time plot revealed a shift in EMA3 after 90 min, indicating a partial relaxation behaviour. The profile suggests an initial delay in polymer chain disentanglement, implying a transition from pure Fickian to relaxation-driven (Case II) diffusion [15]. Furthermore, the *R*/*F* (relaxation to Fickian contribution) *vs.* timescale and *R*/*F vs.* swelling, % plots demonstrated a gradual increase in relaxation contribution, with a negative slope indicating a delayed polymer relaxation phase, particularly at extended time points, as depicted in Figure 7(A). Similarly, the *in vitro* drug release kinetics followed a similar interpretive pattern, showing *n* values of the Korsmeyer-Peppas model closer/nearer to 0.5, consistent with a Fickian-controlled drug release [31].

The Peppas-Sahlin model, however, showed higher values of *k*_1_ compared to *k*_2_, confirming diffusion-based release mechanisms over relaxation-mediated systems [31]. The plots of *R*/*F vs.* time and *R*/*F vs.* drug release, % supported the dominance of a Fickian diffusion system over polymeric relaxation on drug transport. Hence, while the hydration behaviour showed signs of time-dependent partial polymer relaxation, the *in vitro* drug release kinetics remained significantly governed by Fickian diffusion, indicating a stable diffusion-controlled profile from the ocular film matrix, as displayed in Figure 7(B).

The Fickian contribution (*F*), relaxation contribution (*R*), and relaxation over Fickian contribution (*R*/*F*) were calculated from Equations (6) to (8).



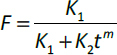

(6)




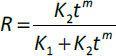

(7)




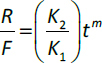

(8)


where *K_1_* is the diffusion constant of swelling/release/permeation kinetics, *K_2_* is the relaxation constant, *t* is the time and *m* is the diffusion exponent.

### Sterility testing

The microorganism growth was observed in the positive control (turbid solution), demonstrating the viability of the media and organisms (as presented in the Supplementary material, Figures S1 and S2). However, no growth of microorganisms was visually evident in the negative control (clear solution), ensuring a sterile environment under aseptic conditions [32]. Subsequently, the absence of growth of microorganisms in the test sample (EMA3) indicated sterility and hence, the sterilized inserts were considered suitable for *in vivo* ocular studies.

### In-vivo intraocular pressure study

**Figure 8.**
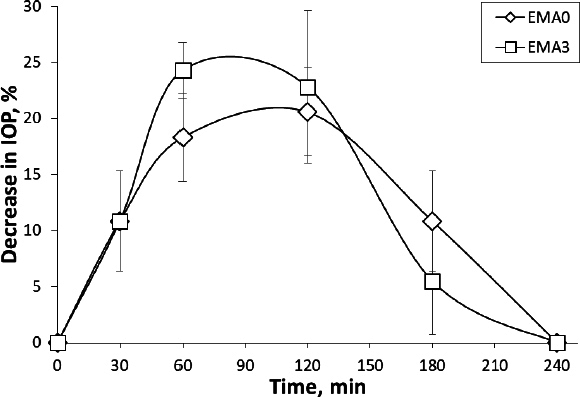
Decrease in intra-ocular pressure with time

The intraocular pressure of a normal eye is around 2.73 ± 0.07 kPa (20.5 ± 0.5 mmHg). Two formulations (EMA0, EMA3) were administered in a normotensive rabbit eye and found that formulation IOP has been reduced (around 20 %) from the normal level 2.67 kPa (20 mmHg) after application of film (EMA0) at 120 min time-point, and the presence of SiO_2_ reduced IOP by more than 24 % at 60 min time-point which can be considered as a clinically significant reduction of IOP as shown in Figure 8 [33].

### In-vivo ocular anti-inflammatory study

**Figure 9.**
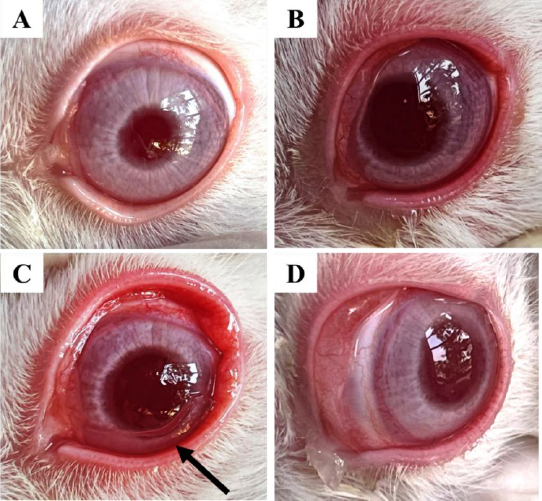
(A) normal eye; (B) carrageenan-induced ocular inflammation with full redness; (C) Fflm (EMA_3_) placed in the cul-de-sac; (D) significantly reduced redness and inflammation after 2.5 h of film administration

Figure 9A shows the normal eye, while Figure 9B shows carrageenan-induced inflammation in the rabbit eye. Acute inflammation is characterized by full redness and watery eyes. Ocular film (EMA3) placed in the cul-de-sac has been hydrated in the tear fluid, as seen in Figure 9C. Within 2.5 h of film administration, redness and inflammation have significantly reduced, as observed in Figure 9D.

### Correlation study

**Figure 10.**
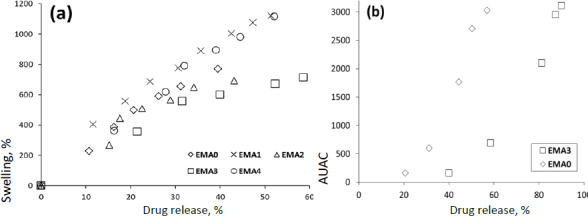
a) Correlation study between drug release and swelling of the film formulation at the same time point, and b) IVIVC between *in vitro* drug release and *in vivo* AUAC of the decreased ocular pressure (EMA0 and EMA3)

A good level of linear correlation has been established between drug release and swelling at the same time point of the film formulation. *In vitro* drug release and *in vivo* area under activity curve (AUC) of the decreased ocular pressure (EMA0 and EMA3) have also been linearly correlated (*in vitro - in vivo* correlation (IVIVC)) [13,34]. These expressive IVIVCs possess both extrapolative and predictive capabilities and will be highly useful for future research. The swelling kinetics of the film could also be estimated from the *in vitro* drug release studies (Figure 10a). Further, the IOP reduction can be predicted from the IVIVC study using *in vitro* drug release data. Hence, the correlation study could help in swelling behaviour of the prepared ocular film as well as the *in vivo* activity of the EMP formulation on normotensive rabbit eyes from the *in vitro* dissolution data of the respective films using linear regression equations [12].

### Stability studies

The accelerated stability studies conducted on the developed ocular film formulation were analysed for FTIR and thermal testing. In the 6^th^ week, the physical characterization of the prepared ocular films showed no significant changes in the FTIR peaks, and the DSC thermogram results also showed no degradation peak [13,34]. The stability study concluded that ocular film formulations demonstrated good stability as evident from Figures S3 and S4.

### Docking study

**Figure 11.**
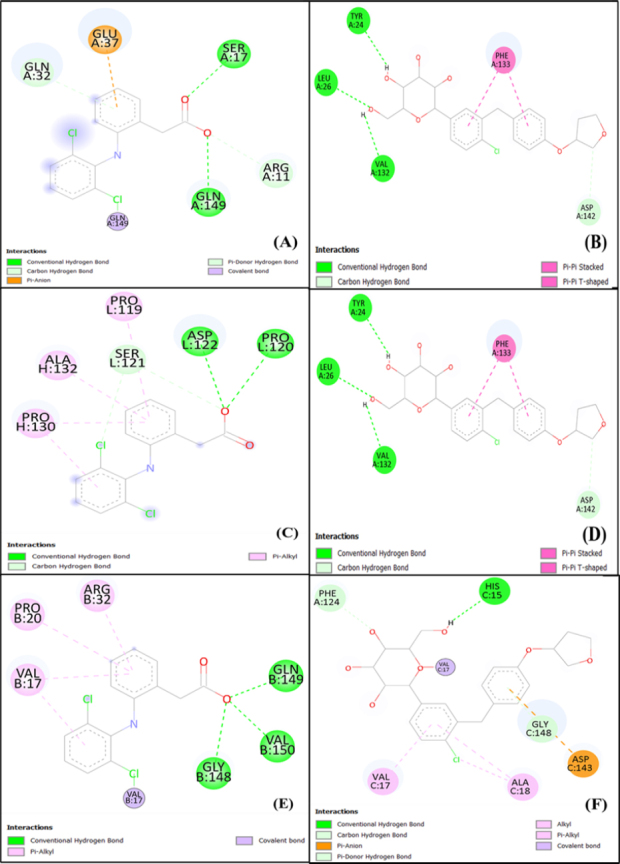
Molecular docking (2D structures) comparison study of diclofenac and empagliflozin with (A, B) IL 1β; (C, D) IL-6; (E, F) TNF-α [A, C, E - diclofenac; B, D, F - empagliflozin]

**Figure 12.**
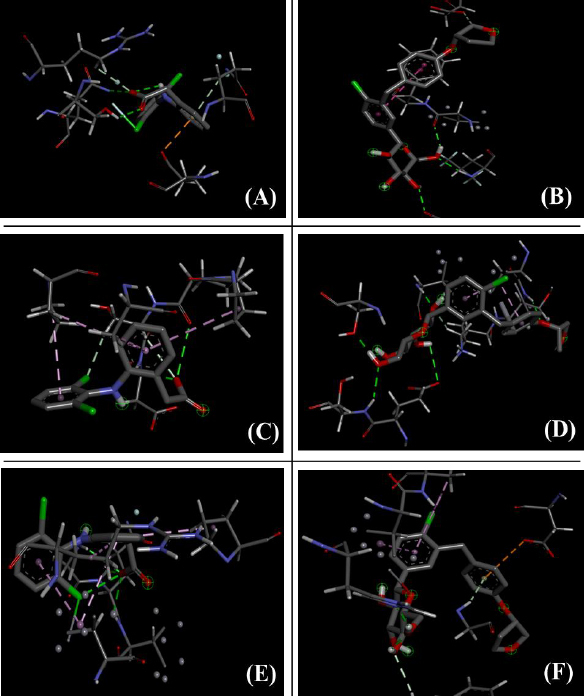
Molecular docking (3D structures) comparison study of diclofenac and empagliflozin with (A, B) IL 1β; (C, D) IL-6; (E, F) TNF-α [A, C, E - diclofenac; B, D, F - empagliflozin]

Figures 11 and 12 display the 2D- and 3D-interaction, respectively, between empagliflozin and inflammatory cytokines such as IL-1β, IL-6 and TNF-α in comparison with the binding of diclofenac with the same receptors.

## Conclusions

An HPMC-based ocular film of empagliflozin was prepared effectively, incorporating colloidal silicon dioxide via casting and solvent evaporation. The highest levels of hydration and swelling were observed with EMA1 and EMA4 (1091 and 986 %, respectively), while EMA2 and EMA3 exhibited minimal hydration (693 and 437 %, respectively). A medium level of hydration was observed in the absence of SiO_2_ (EMA0) of 772 to 792 %, and the behaviour followed the Fickian mechanism by all except EMA3, with partial relaxation. The presence of SiO_2_ in the film resulted in improved diffusion-controlled drug release compared to its absence (EMA0). The presence of SiO_2_ in the film (EMA3) reduced IOP by more than 24 % at 60 minutes and could be considered clinically important in reducing IOP, compared to approximately a 20 % reduction, indicating a hypotensive potential of EMP upon ocular administration of the film without SiO_2_ (EMA0). Meaningful disappearance of redness and inflammation has also been observed using the film EMA3. The docking study further demonstrated that the binding affinity of EMP with IL-1β, IL-6, and TNF-α was more effective than that of diclofenac (a standard NSAID). The correlation study demonstrated a strong relationship between swelling behaviour and drug release, as well as a linear *in vitro-in vivo* correlation (IVIVC) for ocular pressure reduction, with EMA3. Point-to-point correlation established the predictive capability of swelling behaviour as well as the *in vivo* performance of the prepared ocular film on normotensive rabbit eyes from the *in vitro* drug release profile.

## Supplementary material

Additional data are available at https://pub.iapchem.org/ojs/index.php/admet/article/view/2941, or from the corresponding author on request.


