# Supplementary material for: Hypotension and antiphlogistic potential of empagliflozin ocular film: swelling and release kinetics
Source: ADMET DMPK. 2025 Nov 24;14:2941. doi: 10.5599/admet.2941 (PMC12994604; doi:10.5599/admet.2941)
Supplement: Supplementary file 1 [file ADMET-14-2941-S1.pdf]

ADMET & DMPK **14** (2026) S2941

Open Access : ISSN : 1848-7718
http://www.pub.iapchem.org/ojs/index.php/admet

Supplementary material to

Hypotension and antiphlogistic potential of empagliflozin ocular film: swelling and release kinetics

Tanisha Das, Subrata Mallick*, Sourajit Parida, Mouli Das, Rakesh Swain and Sk Habibullah

School of Pharmaceutical Sciences, Siksha 'O' Anusandhan (Deemed to be University), Bhubaneswar, Odisha, India

ADMET & DMPK **14** (2026) 2941; https://doi.org/10.5599/admet.2941

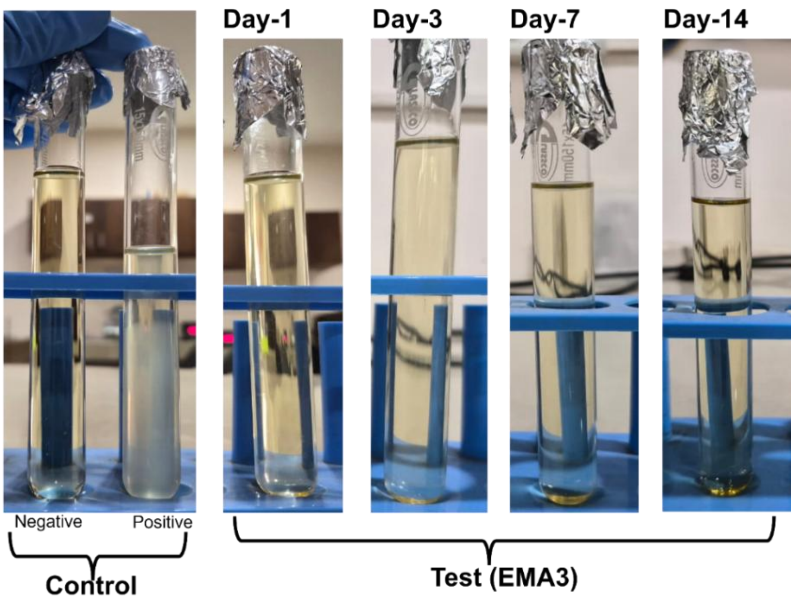

Figure S1. Sterility testing using soyabean casein digest (aerobic) medium

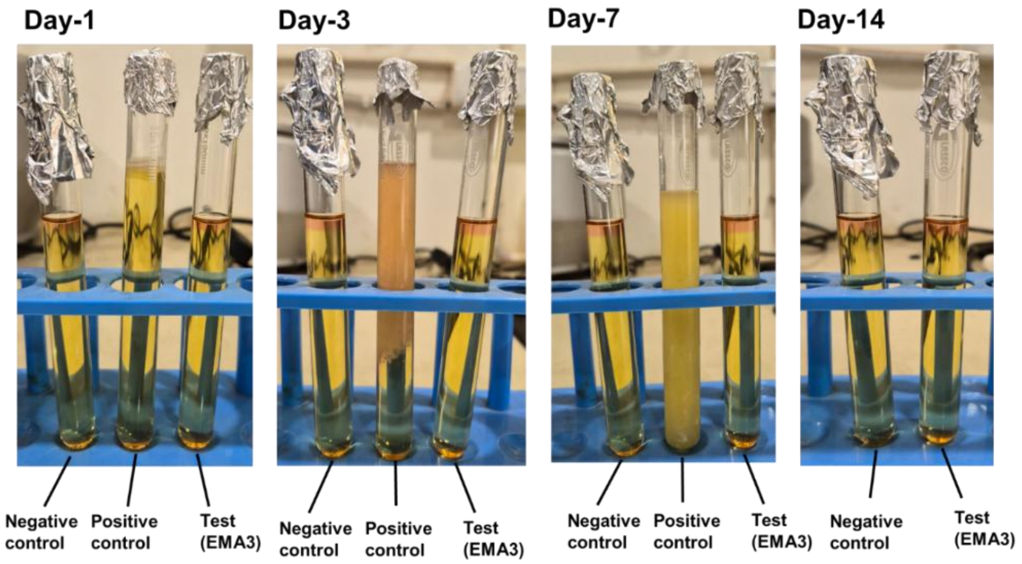

Figure S2. Sterility testing using thioglycollate (anaerobic) medium

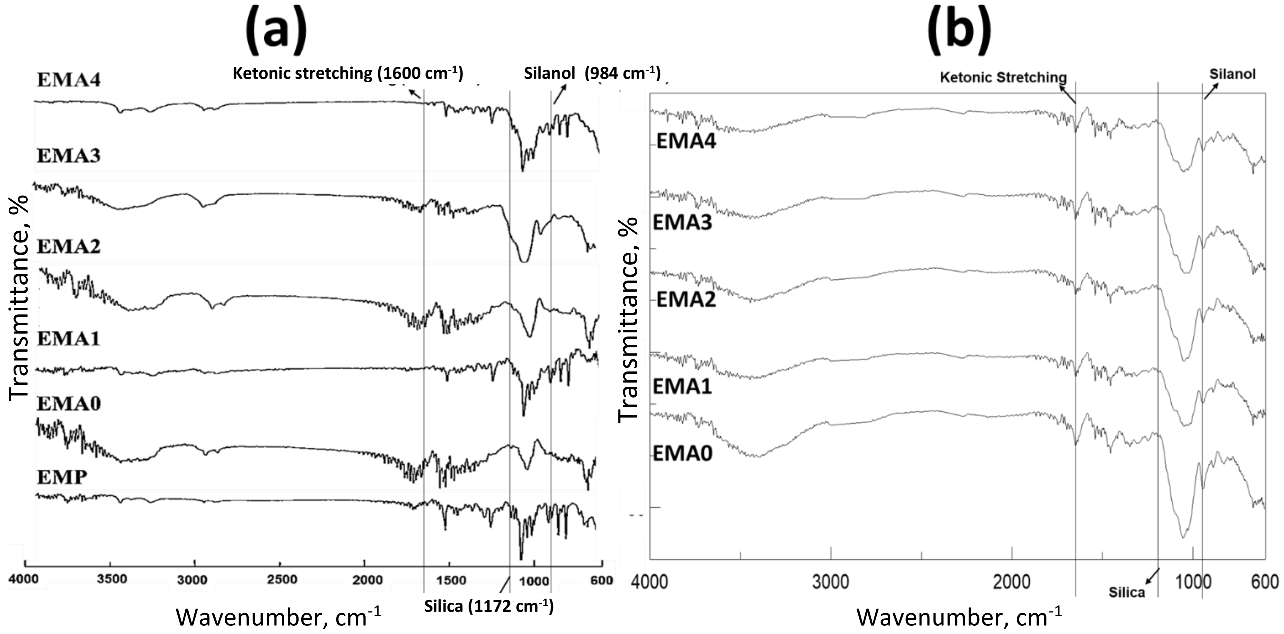

Figure S3: FTIR spectra of EMP-loaded ocular film (a) freshly prepared sample and (b) after 6 weeks of storage at 40 °C and 75 % RH

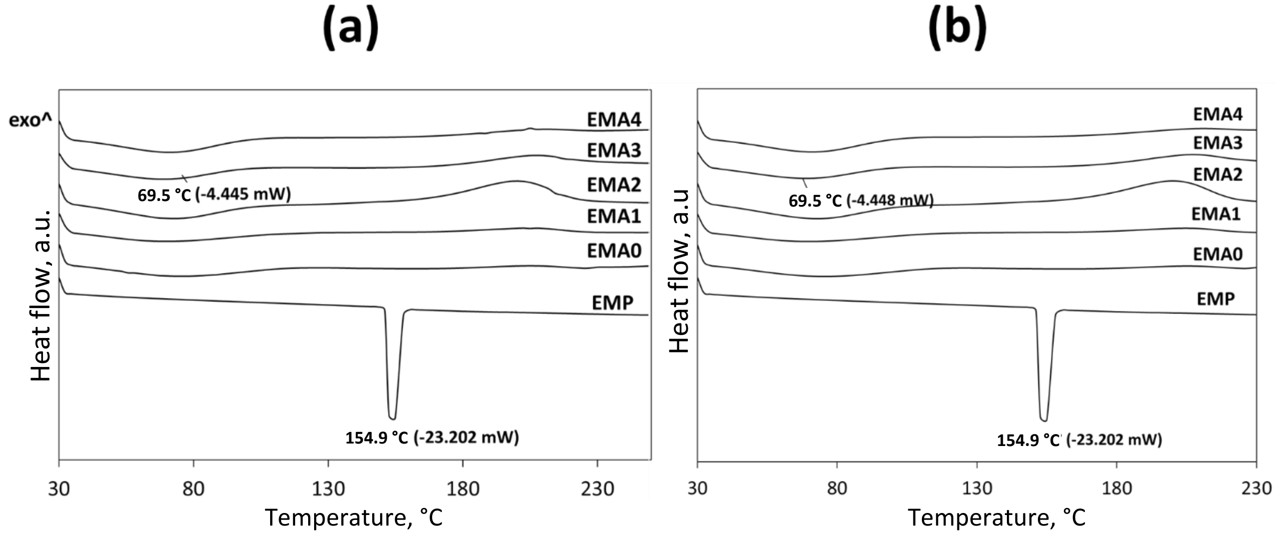

Figure S4. DSC results of pure EMP and its ocular preparations (a) fresh sample and (b) after 6 weeks of storage at 40 °C and 75 % RH

(cc) BY
