# Supplementary material for: Hypotension and antiphlogistic potential of empagliflozin ocular film: swelling and release kinetics
Source: ADMET DMPK. 2025 Nov 24;14:2941. doi: 10.5599/admet.2941 (PMC12994604; doi:10.5599/admet.2941)
Supplement: Supplementary file 2 [file ADMET-14-2941-S1.docx]

ADMET & DMPK **14** (2026) S2941

*
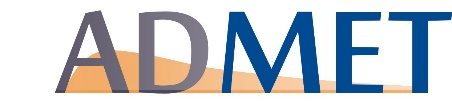
***Open Access : ISSN : 1848-7718**[***http://www.pub.iapchem.org/ojs/index.php/admet***](http://www.pub.iapchem.org/ojs/index.php/admet)

Supplementary material to

**Hypotension and antiphlogistic potential of empagliflozin ocular film: swelling and release kinetics**

Tanisha Das ^[
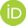
](https://orcid.org/0000-0002-8854-5229)^, Subrata Mallick* ^[
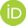
](https://orcid.org/0000-0002-6606-1223)^, Sourajit Parida ^[
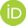
](https://orcid.org/0009-0007-7495-763X)^, Mouli Das ^[
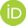
](https://orcid.org/0009-0009-2246-0589)^, Rakesh Swain ^[
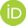
](https://orcid.org/0000-0003-1907-1653)^ and Sk Habibullah ^[
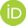
](https://orcid.org/0000-0002-3646-7177)^

School of Pharmaceutical Sciences, Siksha ‘O’ Anusandhan (Deemed to be University), Bhubaneswar, Odisha, India

ADMET & DMPK **14** (2026) 2941; <https://doi.org/10.5599/admet.2941>


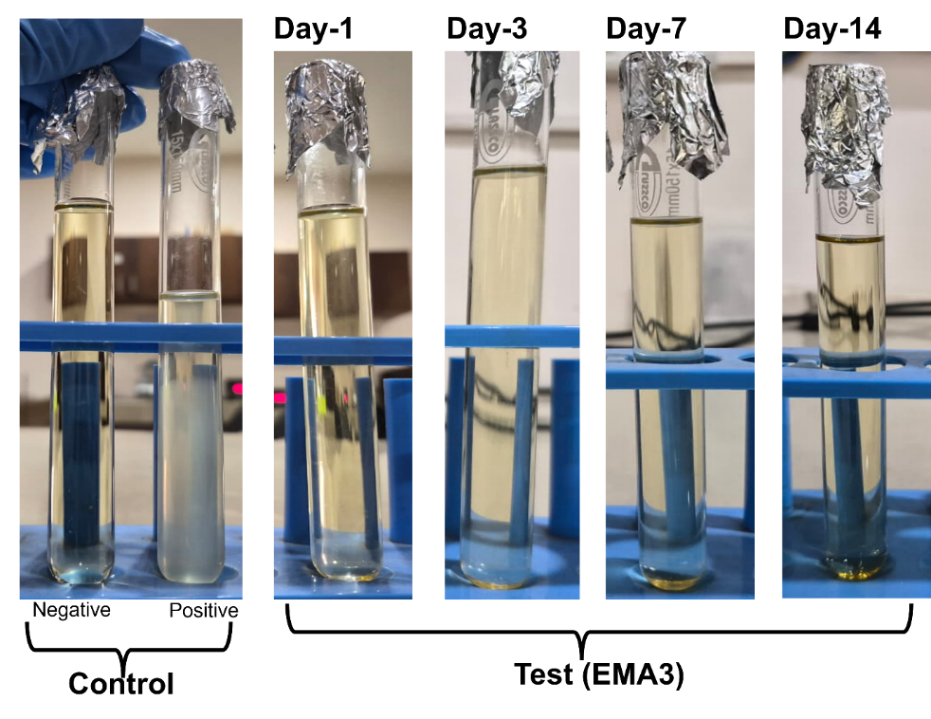


**Figure S1.** Sterility testing using soyabean casein digest (aerobic) medium


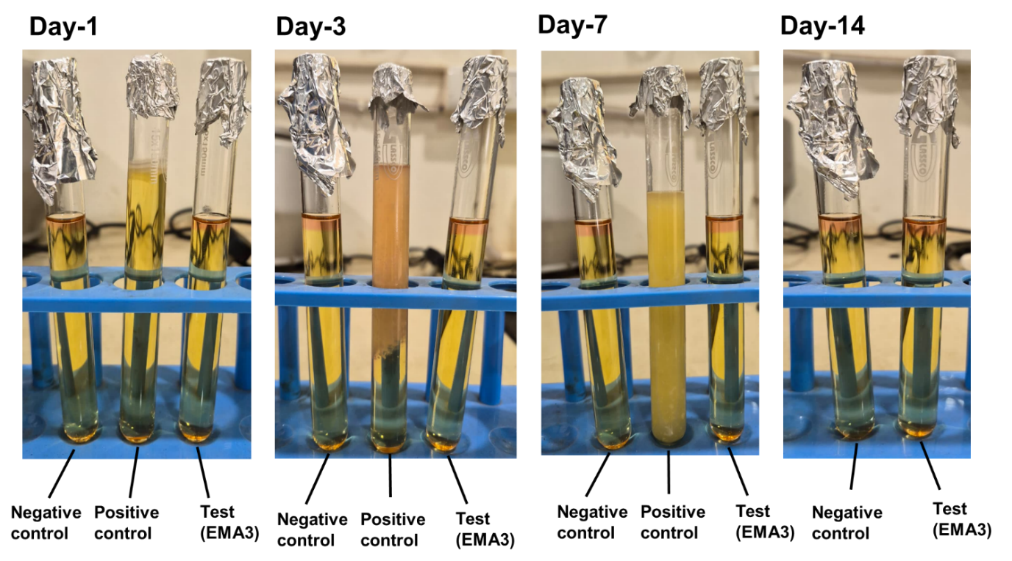


**Figure S2.** Sterility testing using thioglycollate (anaerobic) medium


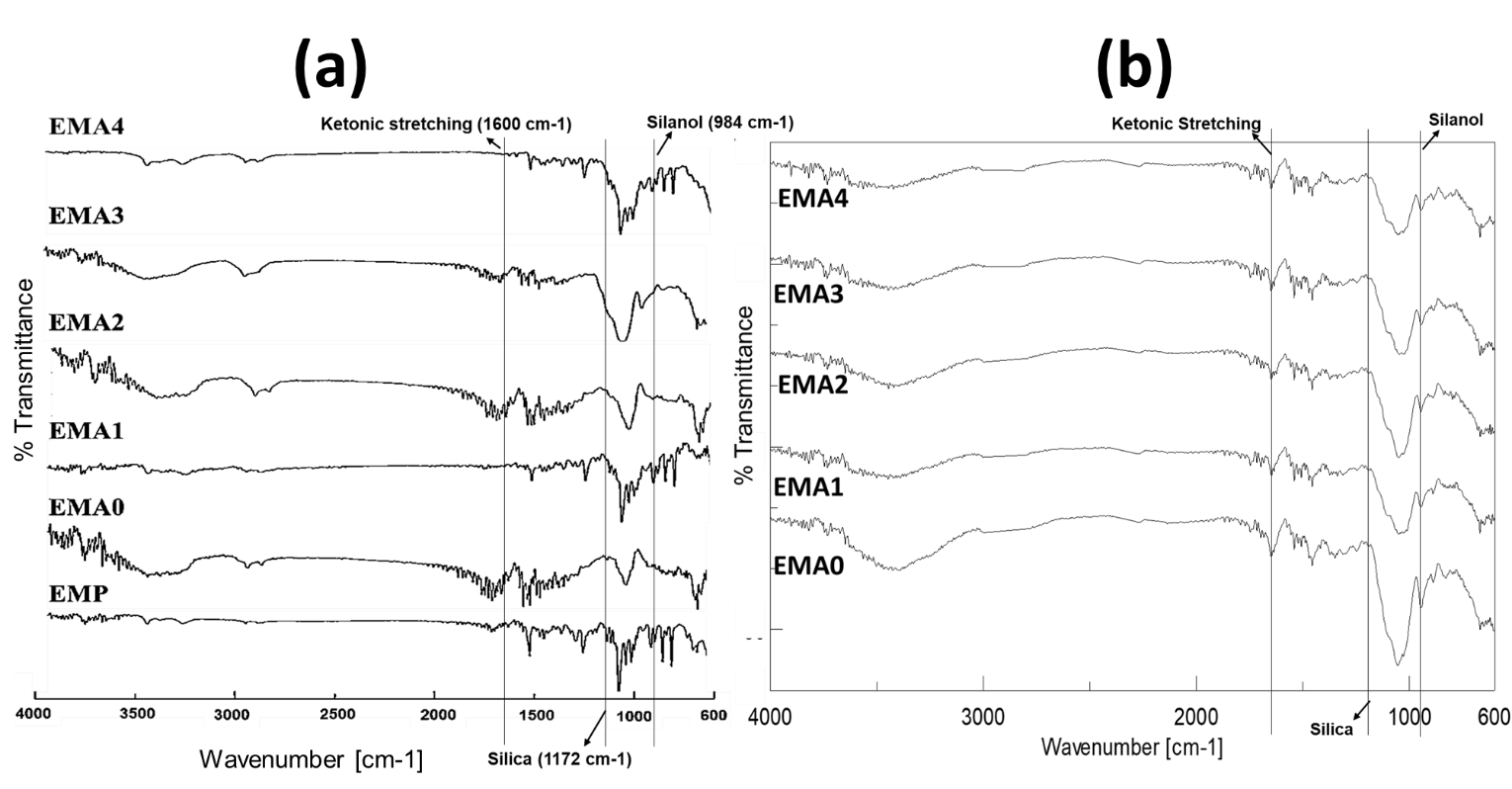


**Silanol (984 cm^-1^)**

**Ketonic stretching (1600 cm^-1^)**

**Silica (1172 cm^-1^)**

Transmittance, %

Transmittance, %

Wavenumber, cm^-1^ Wavenumber, cm^-1^

**Figure S3:** FTIR spectra of EMP-loaded ocular film (a) freshly prepared sample and (b) after 6 weeks of storage
at 40 °C and 75 % RH


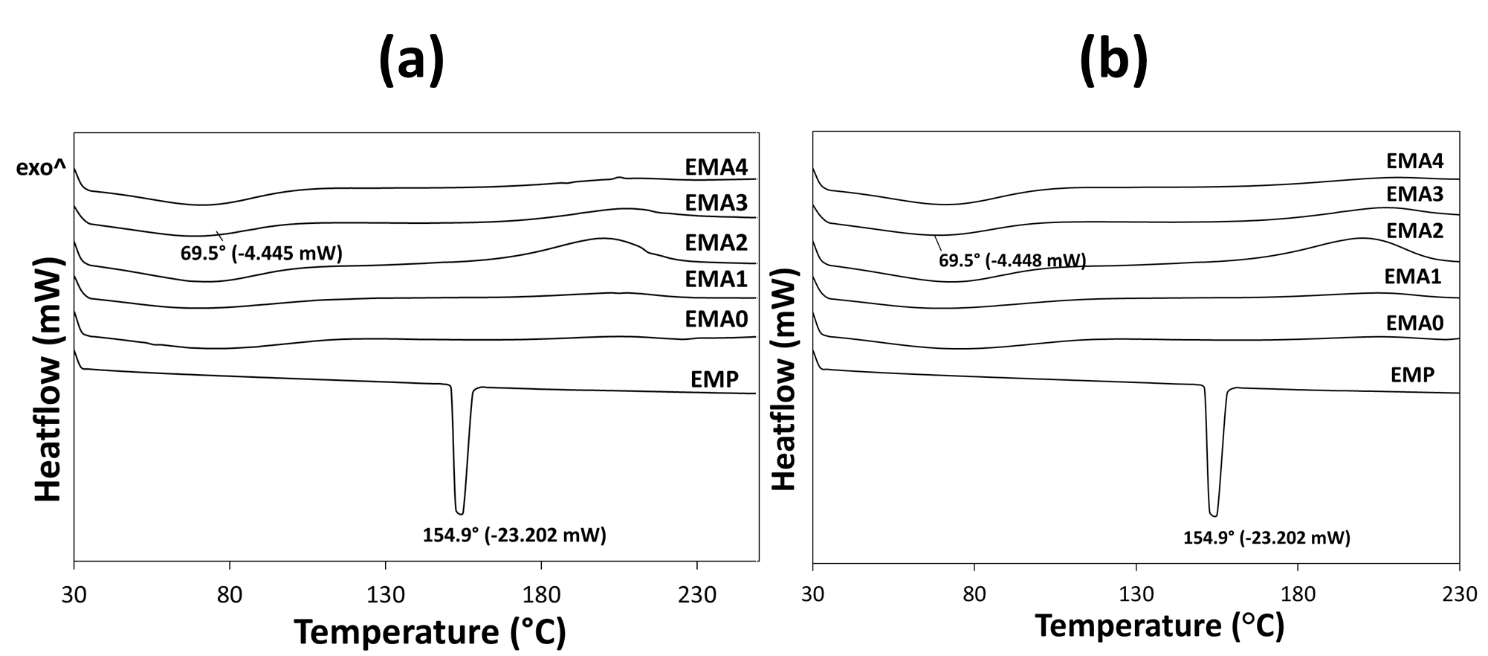


**154.9 °C**

**154.9 °C**

**69.5 °C**

**69.5 °C**

Heat flow, a.u

Heat flow, a.u.

Temperature, °C Temperature, °C

**Figure S4.** DSC results of pure EMP and its ocular preparations (a) fresh sample and (b) after 6 weeks of storage at 40 °C and 75 % RH
